# Improved Chemosensitivity in Metastatic Castration‐Resistant Prostate Cancer: The Synergistic Effects of S‐Adenosylmethionine and Cabazitaxel

**DOI:** 10.1002/cam4.71784

**Published:** 2026-04-13

**Authors:** Roberta Arpino, Francesca Cadoni, Cristina Pagano, Laura Coppola, Vitale Del Vecchio, Aditya Nigam, Laura Mosca, Marina Porcelli

**Affiliations:** ^1^ Department of Precision Medicine University of Campania Luigi Vanvitelli Naples Italy; ^2^ Department of Molecular Medicine and Medical Biotechnology University of Naples Federico II Naples Italy; ^3^ Department of Experimental Medicine University of Campania Luigi Vanvitelli Naples Italy; ^4^ Department of Life Sciences, Health and Health Professions Link University Rome Italy; ^5^ Department of Human Sciences and Promoting of the Quality of Life San Raffaele University Rome Italy

**Keywords:** adjuvant chemoterapy, apoptosis, cabazitaxel, metastatic castration‐resistant prostate cancer, oxidative stress, S‐adenosyl‐L‐methionine

## Abstract

Metastatic castration‐resistant prostate cancer (mCRPC) is the most aggressive kind of prostate cancer (PCa). Because the traditional taxane‐based therapy is ineffective, cabazitaxel (CBZ), a second‐generation, semisynthetic taxane, has been developed. Despite it demonstrating efficacy in patients resistant to docetaxel and paclitaxel, two commonly used taxanes, and representing one of the second‐line therapeutic options for patients with mCRPC, CBZ has limitations, including considerable side issues and reduced drug susceptibility that gradually emerge and constitute the primary cause of therapeutic failure. For the purposes of this study, the application of nature‐derived adjuvants in chemotherapy is emerging as a promising area of research. S‐Adenosyl‐L‐methionine (AdoMet) is a naturally occurring sulfur‐containing nucleoside that serves as the major methyl donor for numerous methyltransferases, which is a key metabolite in the cell, participating in a broad range of essential biochemical processes. In the current research, we bring attention to the effectiveness of the combination of AdoMet and CBZ during treatment of mCRPC cells. Using mCRPC cell lines DU 145 and PC‐3, we found that the combination of CBZ and AdoMet worked better than either agent alone in suppressing cancer cell growth. This synergistic effect may be mediated by increased production of reactive oxygen species (ROS) and a weakening of the cancer cells' antioxidant defenses, including reductions in glutathione, GPX4, and catalase. The resulting oxidative stress caused DNA damage and interference with mitotic spindle assembly, which induces cell cycle arrest and programmed cell death. These data indicate that AdoMet is capable of intensifying CBZ responsiveness in mCRPC cells, making the treatment more effective.

**Abbreviations**
AdoMet: S‐adenosyl‐L‐methionine
ATCC: American Type Culture Collection
BSA: Bovine Serum Albumin
CBZ: Cabazitaxel
CI: Combination Index
CRPC: Castration‐Resistant Prostate Cancer
DRI: Dose Reduction Index
DSBs: DNA double‐Strand Break
ERK1/2: Extracellular signal‐Regulated Kinases 1 and 2
FBS: Fetal Bovine Serum
FDA: Food and Drug Administration
GPX4: Glutathione Peroxidase 4 antibody
GSH: Glutathione
GSSG: Glutathione disulfide
H2AX: H2A histone family member X
JAK: Janus Kinase 2
JNK: c‐Jun N‐terminal Kinase
mAb: monoclonal Antibodies
mCRPC: metastatic Castration‐Resistant Prostate Cancer
MMP‐2: Matrix MetalloPeptidase 2
MTT: 3‐(45‐dimethylthiazol‐2‐yl)‐2,5 diphenyltetrazolium bromide; NAC: N‐acetyl‐l‐cysteine
PARP‐1: poly (ADP ribose) polymerase
PBS: Phosphate‐Buffered Saline
PCa: Prostate Cancer
PSA: Prostate‐Specific Antigen
RIPA: buffer: Radioimmunoprecipitation assay buffer
ROS: Reactive Oxigen Species
RPMI: Roswell Park Memorial Institute
SD: Standard Deviation
SDS‐PAGE: Sodium Dodecyl Sulfate–PolyAcrylamide Gel Electrophoresis
STAT3: Signal Transducer and Activator of Transcription 3
uPA: urokinase‐type Plasminogen Activator
VEGF: Vascular Endothelial Growth Factor

## 1 Introduction

Prostate cancer (PCa) is the most frequently diagnosed male cancer in Western countries with a global number of new cases of approximately 1.6 million per year [1]. Due to its relatively high incidence and number of deaths per year, it represents the fifth most widespread cause of cancer‐related death worldwide [1]. Early diagnosis and timely treatment have significantly improved prognosis; however, advanced PCa, notably castration‐resistant prostate cancer (CRPC), persists as an ongoing therapeutic challenge. Despite progress in understanding tumor biology, managing the disease in its advanced stages remains complex and often ineffective. Traditional therapeutic options include surgery, radiotherapy, and hormone therapy, but chemotherapy and other targeted therapies are required if resistance to these treatments occurs. Taxane‐based chemotherapy has been a cornerstone of advanced PCa treatment since the early 2000s [2]. Among taxanes, cabazitaxel (CBZ) is a second‐generation, semisynthetic analogue of docetaxel, developed to retain therapeutic efficacy in cell lines sensitive to or resistant to docetaxel and paclitaxel, and represents one of the second line treatments for the clinical management of mCRPC [3, 4, 5]. One of the main rationales for its development was to overcome resistance mechanisms associated with docetaxel, particularly its strong binding to proteins resistant to drugs such as the ATP‐dependent efflux pump P‐glycoprotein [5]. CBZ, administered together with prednisone, received FDA approval in 2010 for treating mCRPC in patients with prior docetaxel therapy. While CBZ therapy has exhibited a measurable improvement in survival outcomes in this context, its clinical efficacy remains limited. The response rate of prostate‐specific antigen (PSA) is approximately 39.2%, and disease progression typically occurs after a median of 2.8 months [4]. Moreover, the use of CBZ has shown several limitations, including relevant adverse reactions and the resistance to therapy that gradually emerges constituting the primary cause of therapeutic failure [6]. Complex mechanisms, such as genetic mutations, alterations in cellular signaling pathways, and changes in the tumor microenvironment, assist this resistance. Understanding these mechanisms has stimulated research into alternative therapeutic approaches and combination strategies to enhance chemotherapy efficacy. This highlights the urgent need to overcome innate or acquired resistance to CBZ to improve therapeutic outcomes. Although PCa is among the most widespread cancers affecting men worldwide, the potential of natural compounds has been insufficiently investigated in co‐treatment with conventional antitumoral agents. Notably, natural products have been shown to enhance the effectiveness of chemotherapeutic drugs when co‐administered, potentially offering synergistic effects [7]. Numerous preclinical and limited clinical studies have suggested that some naturally derived substances, such as flavonoids, terpenes, and polyphenols, possess antitumor properties that could increase the therapeutic potential of chemotherapy by reducing the toxicity correlated to traditional treatments. These molecules could act synergistically with chemotherapeutic agents, improving tumor cell sensitivity and promoting their death [8]. Furthermore, some natural molecules can modulate drug resistance mechanisms, offering new therapeutic opportunities to fight advanced PCa. Notably, the combination of CBZ with the natural compound usnic acid was shown to synergistically reduce cell viability and induce apoptosis in mCRPC cells, with reactive oxygen species (ROS) accumulation and disruption of mitochondrial pathways, highlighting the potential of natural products as chemosensitizing agents [9]. Similarly, capsaicin, a naturally occurring molecule present in red peppers, has been reported to enhance the cytotoxic effects of docetaxel in advanced PCa cell models through the modulation of metabolic and apoptotic pathways, involving inhibition of the PI3K/Akt/mTOR axis by taxanes and activation of AMPK by capsaicin [10].

Additionally, thymoquinone combined with docetaxel induced synergistic apoptosis via PI3K/Akt pathway inhibition in PCa cells [11], while rutin and sodium butyrate together reduced viability and increased ROS in mCRPC PC‐3 cells [12]. Moreover, kaempferol enhanced docetaxel‐induced autophagy in vitro and in vivo [13], and combinations of docetaxel with natural polyphenols such as calebin A, 3′‐hydroxypterostilbene, hispolon, and tetrahydrocurcumin augmented the antiproliferative effect [14]. Collectively, these findings support the concept that natural or metabolism‐targeting compounds may serve as effective adjuvants to standard chemotherapy in advanced and castration‐resistant PCa. In addition to mechanistic studies of anti‐cancer agents, it is notable that many patients with cancer choose to use dietary supplements during chemotherapy.

However, the effects of such supplements on chemotherapy efficacy and toxicity remain poorly understood, and clinical evidence for beneficial or deleterious interactions is limited [15].

In this scenario, S‐adenosyl‐L‐methionine (AdoMet) is a naturally occurring sulfur‐containing nucleoside that serves as the major methyl donor for most methyltransferases, plays a central role in cellular metabolism, participating in a broad range of essential biochemical reactions [16, 17]. Recent evidence points to a potential function of AdoMet in regulating tumor metabolism [18, 19, 20], modulating the oxidative stress response [21], and attenuating inflammatory processes [22]. Its antitumor properties may enhance the effectiveness of chemotherapy while mitigating the adverse reactions associated with traditional treatments.

The literature contains a relatively restricted number of studies addressing the effects of AdoMet on PCa, in which it exerts antiproliferative activities across multiple biological processes, together with reduction of cellular proliferation, activation of apoptosis, and suppression of cell migration and invasion [23].

Several studies have shown that AdoMet could suppress tumor‐promoting genes, such as VEGF, uPA, and MMP‐2, resulting in reduction of tumor cell invasion in vitro and tumor growth in vivo. These findings support the hypothesis that hypermethylation therapy could represent a potential strategy for advanced PCa [24]. Furthermore, male Balb/c nu/nu mice inoculated with AdoMet‐treated PC‐3 cells developed tumors of reduced volume compared with vehicle‐treated cells [24]. The potential of AdoMet to suppress tumor cell invasion and migration in PCa cells was also confirmed by Schmidt et al., in a work published in 2016. The authors demonstrated that the anticancer effects of AdoMet were driven by inhibiting active demethylation and triggering the remethylation of proto‐oncogene promoters [25]. Additionally, it has been demonstrated that AdoMet triggered the inhibition of ERK1/2 and STAT3 signal pathways, that play roles in tumor survival, migration, invasion and proliferation of tumor cells [23].

Although existing studies highlight AdoMet's effects in PCa, its molecular mechanism remains not fully understood, and its potential to enhance chemotherapy is still largely unexplored. This is especially important in the context of chemoresistance, which poses a significant challenge in PCa treatment.

Identifying new compounds with antitumor activity and minimal toxicity is crucial to improving PCa treatment strategies. In this study, we highlighted the effectiveness of the combination of AdoMet and CBZ in treating mCRPC cells. Our findings suggest that AdoMet, through its pleiotropic properties, enhances the antitumoral effects of CBZ by targeting two main mechanisms: the intensification of reactive oxygen species (ROS) and mitotic spindle formation. This combined approach not only reduces tumor growth but also significantly increases cell death by inducing a state of oxidative stress that compromises DNA integrity and activates apoptotic mechanisms.

## 2 Materials and Methods

### 2.1 Materials

CBZ (Catalog No. SML2487), NAC (Catalog No. A7250), and 3‐(4,5‐dimethylthiazol‐2‐yl)‐2,5 diphenyltetrazolium bromide (MTT) (Catalog No. M2003) were obtained from Sigma‐Aldrich (St. Louis, MO, USA). Radioimmunoprecipitation assay buffer (RIPA buffer) (Catalog No. AR0105‐100) was purchased from Boster (Pleasanton, CA, USA). Bovine serum albumin (BSA), fetal bovine serum (FBS), Roswell Park Memorial Institute (RPMI) 1640 Medium, phosphate‐buffered saline (PBS), and trypsin–EDTA were bought from Gibco (GIBCO, Grand Island, NY, USA). Tissue culture dishes were purchased from Corning (Corning, NY, USA). AdoMet (Catalog No. B9003S) was purchased from New England Biolabs (dissolved in 5 mM H₂SO₄ and 10% ethanol and filtered). The Annexin V‐fluorescein isothiocyanate (V‐FITC) apoptosis detection kit (Catalog No. 556547) was provided by eBioscience (San Diego, CA, USA). Protein analysis dye reagent concentrate (Catalog No. 5000006) and Trans‐Blot Turbo (Catalog No. 1704158) were bought from Bio‐Rad (Hercules, CA, USA). Monoclonal antibodies (mAb) to poly (ADP ribose) polymerase (PARP‐1) (46D11 #9532), *α*‐tubulin (11H10 #2125S), and β‐actin (8H10D10 #3700) were acquired from Cell Signaling Technology (Danvers, MA, USA). Histone H2AX (sc‐517,336) and p‐Histone H2AX (sc‐517,348) were acquired from Santa Cruz Biotechnology (Dallas, TX, USA). Anti‐Glutathione Peroxidase 4 antibody (GPX4) (ab125066) and Anti‐Catalase antibody (ab209211) were acquired from Abcam (Cambridge, UK). DAPI and Hoechst Nucleic Acid Stains (Catalog No. H3569) for nuclei staining and CellROX Green Reagent for oxidative stress detection (Catalog No. C10444) were purchased from Invitrogen (Thermo Fisher Scientific, Monza, Italy). Horseradish peroxidase (HRP)‐conjugated goat anti‐mouse (GxMu‐003‐DHRPX) and HRP‐conjugated goat anti‐rabbit (GtxRb‐003‐DHRPX) secondary antibodies were purchased from Jackson ImmunoResearch Laboratories Inc. (Raleigh, NC, USA). All buffers and solutions were prepared with ultra high‐quality water and were of the purest commercial grade.

### 2.2 Cell Cultures

DU 145 (HTB‐81) and PC‐3 (CRL‐1435) were obtained from the American Type Culture Collection (ATCC, Manassas, VA, USA). They were cultured in RPMI medium 1640 supplemented with 10% FBS, 2 mM L‐glutamine, 50 U/mL penicillin–streptomycin, and 0.1% PlasmocinTM prophylactic (InvivoGen, San Diego, CA, USA) and maintained at 37°C in a humidified atmosphere containing 5% CO_2_. The passages of the cells were kept under 15 for DU 145 and under 20 for PC‐3. All the cell lines were authenticated using Short Tandem Repeat (STR) analysis as described in 2012 in ANSI Standard (ASN‐0002), with DU 145 showing more than 80% match and PC‐3 showing 100% match for the corresponding ATCC human cell lines. All cell lines were mycoplasma free prior to use in the experiments.

### 2.3 Cell Viability Assay

The effect of AdoMet both on cell viability and on antiproliferative activity of CBZ was assessed by an MTT assay according to the manufacturer's instructions. DU 145 and PC‐3 cells were plated in serum‐enriched media in 96‐well plates at the proper density and treated with fresh medium containing increasing concentrations of AdoMet (from 62.5 to 1000 μM) alone or in combination with CBZ for 24, 48, and 72 h. The incubation medium was then removed, and MTT solution was added in PBS to a final concentration of 0.5 mg/mL. The cells were then incubated at 37°C for 4 h, and the MTT‐formazan crystals were solubilized in 1‐N‐isopropanol/hydrochloric acid 10% solution at 37°C on a shaker for 20 min. The absorbance values of the solution in each well were detected at 570 nm by using a Bio‐Rad IMark microplate reader (Bio‐Rad Laboratories, Milan, Italy). All experiments were performed in quadruplicate. Cell viability was expressed as the percentage of absorbance values of treated samples compared to the control (100%). The IC50 values were calculated by using linear regression analysis. To verify the pivotal role of ROS in the viability of DU 145 and PC‐3 cells treated with AdoMet and CBZ, we also conducted this protocol following a 1 h pre‐treatment with N‐acetyl‐L‐cysteine (NAC), an antioxidant that reduces ROS production, before replacing the medium with the drug treatments.

### 2.4 Drug Combination Studies

For the study of the synergism on growth inhibition, DU 145 and PC‐3 cells were seeded in 96‐multiwell plates at proper density. After 24 h incubation at 37°C the cells were treated with increasing concentrations of AdoMet (from 62.5 to 1000 μM) and CBZ (from 0.087 to 1.5 nM and from 0.175 to 3 nM, respectively) alone or in combination for 72 h, and cell viability was analyzed by MTT assay. Drug combination studies were based on concentration–effect curves generated as a plot of the fraction of unaffected (surviving) cells versus drug concentration [16]. Assessment of synergy was performed by Calcusyn computer program (Biosoft, Ferguson, MO). Combination index (CI) values of < 1, 1, or > 1 indicate synergy, additivity, or antagonism, respectively [17]. The Dose Reduction Index (DRI) represents the measure of how much the dose of each drug, in a synergistic combination, may be reduced at a given effect level compared with the dose of each drug alone.

### 2.5 Flow Cytometry Analysis of Apoptosis

Apoptosis was evaluated by flow cytometry by using Annexin V‐FITC in conjunction with a vital dye PI to distinguish apoptotic (Annexin V‐FITC‐positive, PI‐positive) from necrotic (Annexin V‐FITC‐negative, PI‐positive) cells. DU 145 and PC‐3 cells were seeded in 6‐well plates at a density of 5 × 10^4^ cells/well and incubated in the presence of AdoMet 400 μM and CBZ 0.7 nM and 1.5 nM, respectively. After 72 h, cells were harvested by incubation with trypsin–EDTA, washed with PBS twice, and collected by centrifugation. The cells were then resuspended in 200 μL of Binding Buffer 1× and incubated with 2 μL Annexin V‐FITC and 2 μL PI (20 μg/mL) for 30 min at room temperature, as recommended by the manufacturers. The determination of viable cells and early apoptotic, late apoptotic, and necrotic cells was carried out with a BD FACSCanto II System (Becton & Dickinson, Mountain View, CA, USA). For each sample, 20,000 events were recorded. Analysis was carried out by triplicate determination on at least three separate experiments. Data were analyzed using FlowJo V10 software (FlowJo LLC, USA).

### 2.6 Protein Extraction and Western Blotting Analysis

DU 145 and PC‐3 cells were cultured in 6‐well plates at a density of 10 × 10^4^ cells/well and 5 × 10^4^ incubated in the presence of AdoMet 400 μM and CBZ 0.7 nM, and AdoMet 400 μM and 1.5 nM, respectively, for 72 h and then processed for Western blotting analysis. Briefly, after treatment, the cells were harvested, lysed on ice for 40 min, centrifuged at 18,000 × g in an Eppendorf microcentrifuge for 30 min at 4°C, and the supernatant was collected. Protein concentration was determined and compared with the BSA standard curve. Equal amounts of sample proteins were separated by sodium dodecyl sulfate–polyacrylamide gel electrophoresis (SDS‐PAGE) and electrotransferred onto nitrocellulose membranes by Trans‐Blot Turbo (Bio‐Rad). The membranes were washed in 1 mM Tris–HCl, pH 8.0, 150 mM NaCl, and 0.05% Tween 20 (TBST), blocked with TBST supplemented with 5% nonfat dry milk, and incubated first with specific primary antibodies overnight at 4°C in TBST and 5% nonfat dry milk and then with HRP‐conjugated secondary antibodies. All primary antibodies were used at a dilution of 1:1000 and 1:500; all secondary antibodies were used at a dilution of 1:5000. The immunoblots were then developed using enhanced chemiluminescence detection reagents ECL (Cyanagen, Bologna, Italy) and detected using the Azure 400 Visible Fluorescent Imager (Azure Biosystems Inc., Dublin, CA, USA) and ChemiDoc XRS+ System (Bio‐Rad). All immunoblots were analyzed with ImageJ software 1.48 (National Institutes of Health, Bethesda, MD, USA).

### 2.7 Determination of ROS by the CellROX Assay

DU 145 and PC‐3 cells were seeded in 6‐well plates at the proper density, and after 24 h of incubation, the cells were treated with AdoMet 400 μM and CBZ 0.7 nM and 1.5 nM for 72 h. The positive control was represented by the cells treated with menadione at the final concentration of 100 μM for 1 h at 37°C. After treatment, the cells were treated with 5 μM CellROX green reagent by adding the probe to the complete media and incubating at 37°C for 30 min. Subsequently, cells were washed with PBS and observed by fluorescence microscopy using the EVOS FL Cell Imaging System (Thermo Scientific, Rockford, USA), where snapshot images were captured to examine the ROS production. Cell fluorescence intensity was then analyzed with a BD FACS Canto II System. Briefly, the cells were detached by incubation with trypsin–EDTA, washed twice with PBS, and collected by centrifugation. For each sample, 20,000 events were acquired. Analysis was carried out by triplicate determination in at least three separate experiments. Data were analyzed using FlowJo V10 software.

### 2.8 Reduced Glutathione Assay

The intracellular glutathione (GSH) content was assessed and optimized according to Perrone et al. [26]. The cells, after treatment with AdoMet 400 μM and CBZ 0.7 nM and AdoMet 400 μM and 1.5 nM for 48 and 72 h, were detached by incubation with trypsin–EDTA, washed twice with PBS, and collected by centrifugation. Cell pellets were lysed with 0.4 mL of lysis buffer for 10 min, and the proteins were precipitated by the addition of 0.4 mL of a cold solution of metaphosphoric acid (1.67 g metaphosphoric acid, 0.2 g EDTA, and 30 g NaCl in 100 mL water). The samples were placed at 4°C for 10 min, and a subsequent centrifugation at 18,000 × *g* for 10 min removed the precipitated proteins. Finally, 0.45 mL of supernatant was mixed with an equal volume of 0.3 M Na_2_HPO_4_. To determine the reduced GSH, 0.1 mL of a DNTB solution (20 mg DTNB plus 1% sodium citrate in 100 mL of water) was added to the solution. After 10 min of incubation at room temperature, the absorbance of the samples was measured at a wavelength of 412 nm.

### 2.9 Immunofluorescence Analysis

To perform immunofluorescence analysis, 25 × 10^4^ cells/cm^2^ of DU 145 and 15 × 10^4^ PC‐3 cells were grown on glass cover slips placed in 24‐well culture plates. After treatment with AdoMet 400 μM and CBZ 0.7 nM and 1.5 nM for 72 h, cells were fixed with 4% paraformaldehyde, permeabilized with 0.2% Triton×‐100, and blocked using PBS‐BSA 0.4%. Cells were incubated with specific primary antibodies (anti‐p‐Histone‐H2AX and anti‐*α*‐tubulin to evaluate the number of H2AX foci and the mitotic spindle assembly respectively) at a dilution of 1:200 overnight at 4°C. Cells were then rinsed three times with PBS 1× and hybridized with a secondary antibody coupled to Alexa Fluor 488 (Jackson ImmunoResearch, Cambridge, UK) or Dylight 594 (Abcam, Cambridge, UK) for 1 h at room temperature. Cellular nuclei were counterstained using 2.5 μg/mL Hoechst‐33,258 for ~5 min. Coverslips were then mounted on glass slides. Microscopy images were obtained using a Zeiss LSM 700 laser scanning confocal microscope equipped with a plan apochromat 63× (NA 1.4) oil immersion objective. The quantification of H2AX foci was performed by using ImageJ software 1.48. Approximately 100 nuclei for each treatment group were scored in each experiment, and a threshold of 5 foci per cell was considered positive.

### 2.10 Statistical Analysis

Statistical analysis was carried out with the GraphPad Prism 7.0 software for Windows (GraphPad Software Inc., San Diego, CA, USA). Data are expressed as mean ± standard deviation (SD) and analyzed for statistical significance using the two‐tailed Student's *t*‐test. For multiple comparisons, ANOVA analysis was used, followed by Bonferroni correction. The *p*‐values *p < 0.05, **p < 0.01, ***p < 0.005, and ****p < 0.001 were considered statistically significant. All experiments were repeated at least 3 times and performed in triplicate.

## 3 Results

### 3.1 AdoMet Causes Concentration‐ and Time‐Dependent Growth Inhibition in mCRPC Cells

**FIGURE 1:**
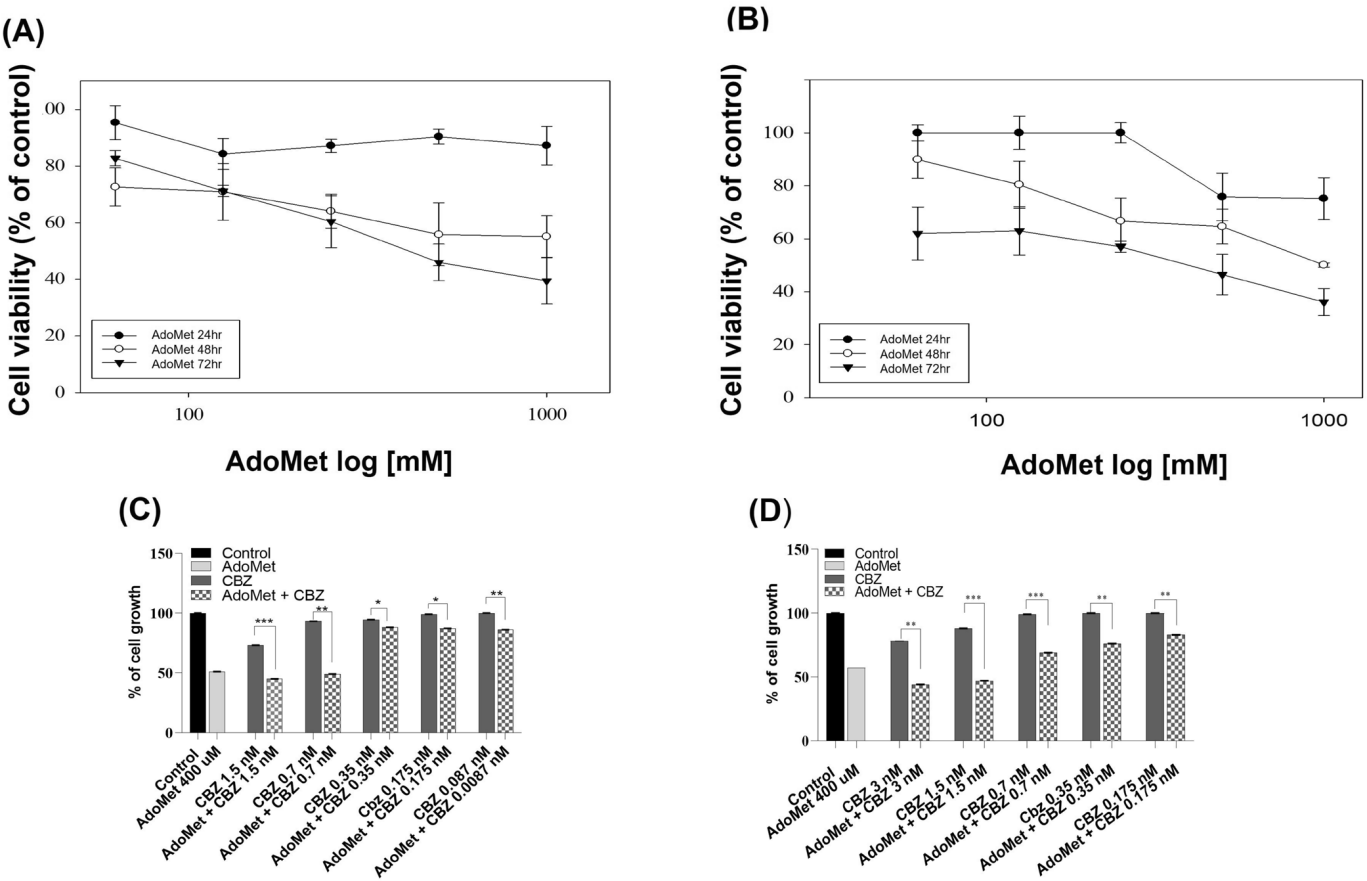
Effect of AdoMet on mCRPC cells viability and its antiproliferative synergistic activity with different concentrations of CBZ. (A) DU 145 and (B) PC‐3 cells were treated or not (control) with increasing amounts of AdoMet (62.5–1000 μM) for 24, 48 and 72 h, then cell viability was assessed by MTT assay. (C) DU 145 and (D) PC‐3 cells were treated or not (control) with increased amounts of CBZ alone or in combination with AdoMet 400 μM for 72 h, then cell viability was assessed by MTT assay. Data represent the average of three independent experiments; error bars depict the standard deviation (SDs) (*p < 0.05, **p < 0.01, ***p < 0.001).

To investigate the antiproliferative properties of AdoMet, we initially assessed the influence of this compound on DU145 and PC‐3 cell viability. As depicted in Figure 1A,B, treatment with escalating doses of AdoMet (62.5–1000 μM), administered for increasing durations, resulted in a statistically significant reduction in cell survival, exhibiting both time‐ and dose‐dependence. MTT analysis revealed IC₅₀ values of approximately 400 μM at 72 h for both DU145 and PC‐3 cells. AdoMet demonstrated efficacy in inhibiting mCRPC cell proliferation consistent with findings reported in other human cancer cell types, including colorectal, liver and breast cancers [19, 27, 28].

### 3.2 AdoMet Potentiates the CBZ Antitumor Effects in mCRPC Cells

We next explored the antitumor effect of AdoMet combined with CBZ by performing drug combination studies using CalcuSyn software (Table 1). Cells were treated with progressively increasing, equipotent concentrations of AdoMet and CBZ for 72 h, which resulted in strong synergistic interactions.

**TABLE 1:** Combination index (CI) and dose reduction index (DRI) values for AdoMet and CBZ combinations in DU 145 and PC‐3 cell lines.

Fa	CI (Mutually exclusive)	CI (Mutually non‐exclusive)	DRI AdoMet	DRI CBZ
DU 145				
0.37	0.260	0.277	8.937	6.751
0.46	0.667	0.776	3.504	2.618
0.63	0.584	0.667	4.052	2.964
PC‐3				
0.49	0.312	0.332	11.01	4.52
0.55	0.507	0.561	6.51	2.83
0.59	0.881	1.047	3.65	1.65

*Note:* CIs smaller than 0.8 indicate strong synergism; CIs smaller than 0.9 indicate sinergysm; additivity between 0.9 and 1.2 or antagonism more than these values. DRI values represent the order of magnitude (fold) of dose reduction obtained for IC (DRI) in combination setting compared with each drug alone.

In detail, co‐treatment with AdoMet and CBZ exhibited a synergistic effect in DU 145 cell line, with a CI < 1 for Fa values ≤ 0.7. Fa value represents the fraction of the population that is affected by the treatment, showing the percentage of cell inhibition achieved by the drug combination. CI is a key parameter in the Chou–Talalay method [29] and quantifies the degree of interaction between two drugs. It compares the effect of the drug combination to the effect of each drug alone. Notably, with Fa = 0.37, the CI was 0.26 (mutually exclusive), indicating strong synergy. DRI quantifies how much the dose of one drug can be reduced when combined with another drug while maintaining the same level of efficacy. The DRI values support an efficient dose reduction for CBZ up to 6.7‐fold and reveal substantial reductions in drug doses. In PC‐3 cell line, the combination of AdoMet and CBZ demonstrated significant synergy for Fa values between 0.49 and 0.55 (CI of 0.312 and 0.507, respectively). The DRI values confirm an effective dose reduction, up to 4.52‐fold for CBZ. These results represent the first demonstration of the synergistic interaction between AdoMet and CBZ in inhibiting cell proliferation.

To assess the optimal concentration to use for defining the biological mechanism by which AdoMet sensitizes mCRPC cells to the effect of CBZ, we performed a proliferation assay using decreasing doses of CBZ (ranging from 1.5 to 0.0087 nM for DU 145 cells and from 3 to 0.175 nM for PC‐3 cells) either alone or combined with 400 μM of AdoMet for 72 h. Based on the data presented in Figure 1C,D, we selected the following combinations for further investigation depending on the cell line: 400 μM AdoMet and 0.7 nM CBZ for DU 145 cells, and 400 μM AdoMet and 1.5 nM CBZ for PC‐3 cells.

### 3.3 AdoMet and CBZ Combination Reduced Antioxidant Defenses in mCRPC Cells

**FIGURE 2:**
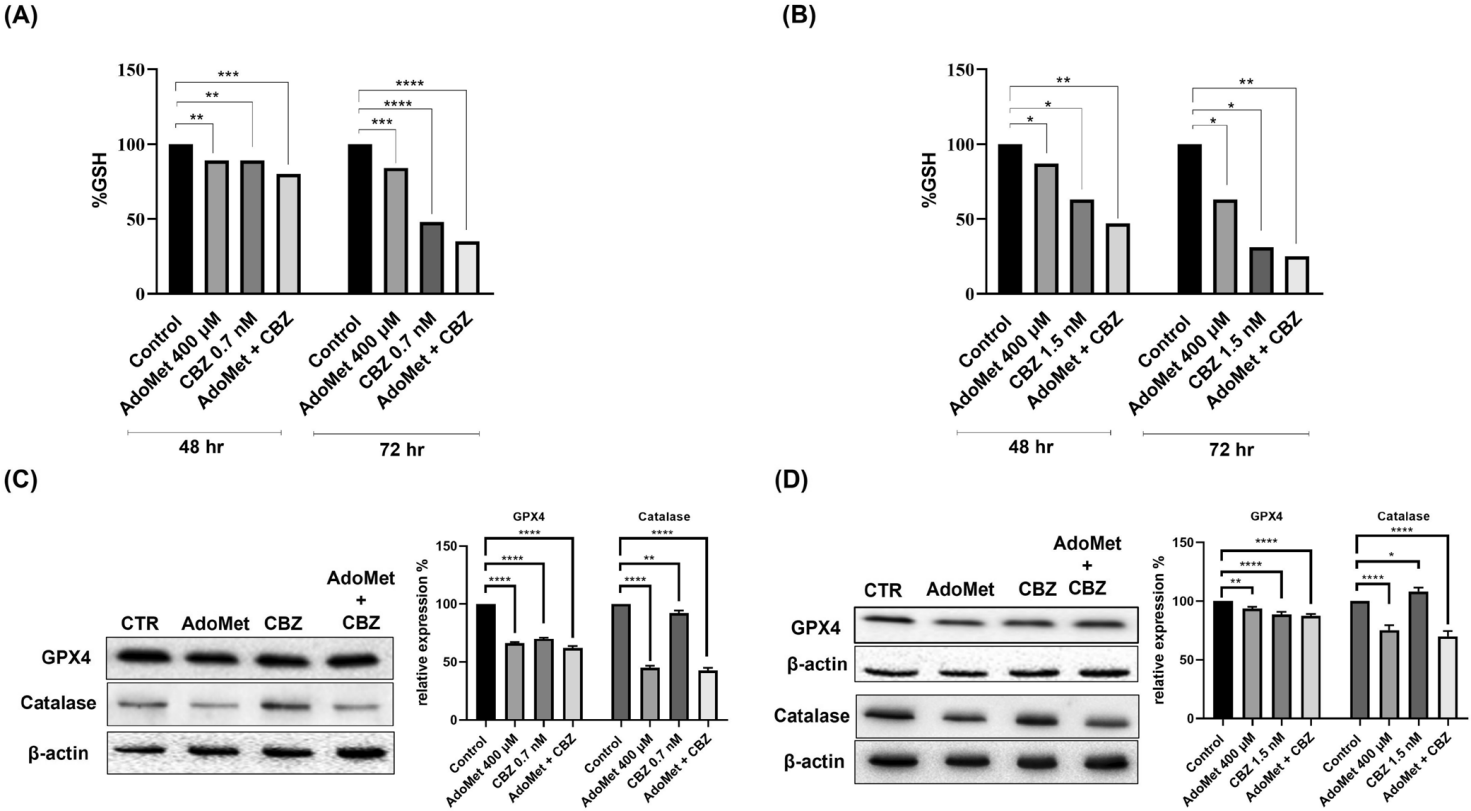
Effect of AdoMet, CBZ and their combination on GSH decrease in mCRPC cells. (A) DU 145 and (B) PC‐3 were treated or not (control) with 400 μM of AdoMet with 0.7 nM and 1.5 nM of CBZ respectively, alone or in combination for 48 and 72 h. Statistical analysis was performed with one‐way ANOVA. Values represent the means of three experiments ± SD (* *p* < 0.05, **p < 0.01, ***p < 0.001, ****p < 0.0001). The protein content of GPX4 and catalase in (C) DU 145 and (D) PC‐3 cells was detected by Western blot analysis using the total cell lysates. The house‐keeping protein β‐Actin was used as loading control. The graph shows the relative densitometric analysis, expressed as percentage of untreated control (100%). Error bar represents the standard deviation, ****p < 0.0001 versus untreated cells. The images are representative of three immunoblotting analyses that were obtained from three independent experiments.

GSH is a key component in the cellular antioxidative defense. Its high concentration in tumor cells supports the neutralization of ROS and detoxification of xenobiotics, making it a potential target for anticancer therapy [30]. A substantial body of evidence indicates that the loss of intracellular GSH increases the sensitivity of cancer cells to chemotherapeutic compounds and oxidative stress. Figure 2 shows the percentage of GSH measured after treatment with 400 μM AdoMet and 0.7 nM CBZ (Figure 2A) and with 400 μM AdoMet and 1.5 nM CBZ (Figure 2B) alone or in combination after 48 and 72 h in DU 145 and in PC‐3 cells, respectively. The GSH level measured after treatment with the combined drugs was significantly lower than that observed in both control cells and cells treated with a single agent. Moreover, the effects were more pronounced after 72 h, above all in DU 145 cells. The significant decrease in GSH levels indicates that the combined treatment effectively disrupts the antioxidant defenses of mCRPC cells, thereby increasing their susceptibility to oxidative damage. Moreover, as indicated by Western blot analysis, AdoMet, CBZ, and their combination cause the reduction of the level of expression of antioxidant enzyme involved in counteracting oxidative stress, preserving redox homeostasis, and protecting cells from ROS‐mediated damage, such as Anti‐Glutathione Peroxidase 4 antibody (GPX4) and catalase (Figure 2C,D).

These findings suggest that AdoMet and CBZ, by modulating the cellular redox balance, might provide a potential approach to enhance tumor cells' sensitivity to oxidative stress and improve the efficacy of antitumor treatments.

### 3.4 AdoMet and CBZ Increase ROS Levels in mCRPC Cells

**FIGURE 3:**
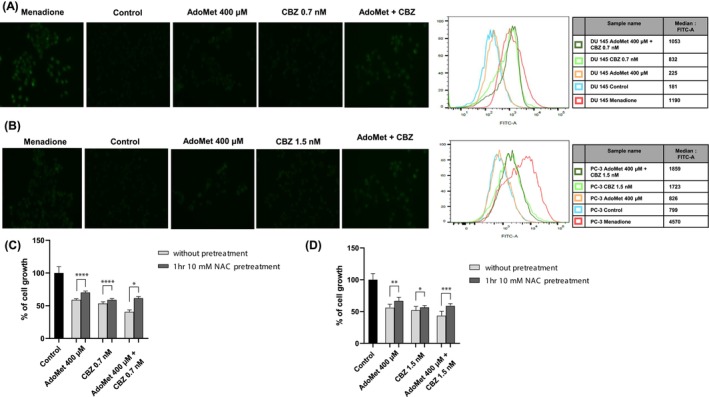
Effects of AdoMet, CBZ, and their combination on ROS accumulation in mCRPC cells. (A) DU 145 and (B) PC‐3 cells were treated or not (control) with 400 μM of AdoMet alone or in combination with 0.7 nM and 1.5 nM of CBZ respectively for 72 h; then they were incubated with CellROX green and analyzed by flow cytometry and fluorescence microscopy. Menadione was used as positive control. The FlowJo software was used to calculate median fluorescence to measure ROS levels. At least 2 × 10^4^ events were acquired in log mode. Analysis was carried out by triplicate determination on at least three separate experiments. (C) DU 145 and (D) PC‐3 cells were pretreated or not with N‐acetyl‐L‐cysteine (NAC, 10 mM) for 1 h and subsequently treated with AdoMet 400 μM, CBZ (0.7 nM for DU 145 or 1.5 nM for PC‐3), or their combination for 72 h. Cell viability was assessed by MTT assay and expressed as percentage of cell growth relative to untreated control cells (control), set to 100%. White bars represent cells without NAC pretreatment, whereas gray bars indicate NAC‐pretreated cells. Data are reported as mean ± SD of at least three independent experiments performed in triplicate (* *p* < 0.05, **p < 0.01, ***p < 0.001 and ****p < 0.0001).

Overproduction of ROS causes cellular damage and participates in the regulation of multiple biological key processes, such as autophagy, apoptosis, mitochondrial disruption, endoplasmic reticulum stress, and DNA damage [31]. Exogenous compounds capable of inducing ROS production or accumulation represent an important strategy in ROS‐based cancer therapies. Recently, the amelioration of oxidative stress in tumor cells by generating ROS and reducing GSH was reported to achieve superior antitumor efficacy [32]. In this work, it is evaluated ROS concentration within mCRPC cell lines following treatment with 400 μM AdoMet and 0.7 nM CBZ (Figure 3A) and with 400 μM AdoMet and 1.5 nM CBZ (Figure 3B) alone or in combination for 72 h in DU 145 and in PC‐3 cells, respectively. Intracellular ROS accumulation was assessed by flow cytometry employing a cell ROX‐based assay, a cell permeant dye that is weakly fluorescent and exhibits bright green photostable fluorescence upon oxidation by ROS. We observed that in DU 145 cells the ROS levels induced by the single drugs were approximately the same as those detected in the untreated cells, while approximately a 1.6‐fold rise in ROS intensification was detected when the two drugs were used in combination (Figure 3A). In the PC‐3 cells, the AdoMet treatment induces a slight increase in ROS levels, while CBZ induces a strong rise of ROS that was more evident in combined treatment, showing a 2.4‐fold increase if analyzed to control cells (Figure 3B). Our findings show that combining AdoMet and CBZ notably enhances ROS production in mCRPC cells, suggesting that the therapeutic strategy effectively increases oxidative stress within the cells. Moreover, to assess the pivotal role of ROS in the regulation of DU 145 and PC‐3 cell viability following AdoMet and CBZ treatment, cell viability was evaluated after a 1 h pretreatment with NAC. As shown in Figure 3C,D, NAC‐mediated suppression of ROS production significantly restored cell viability compared with cells exposed to the same treatments in the absence of NAC. These findings indicate that ROS accumulation induced by AdoMet and CBZ critically contributes to the impairment of survival and proliferation of mCRPC cells.

### 3.5 AdoMet and CBZ Synergistically Induce DNA Damage in mCRPC Cells

**FIGURE 4:**
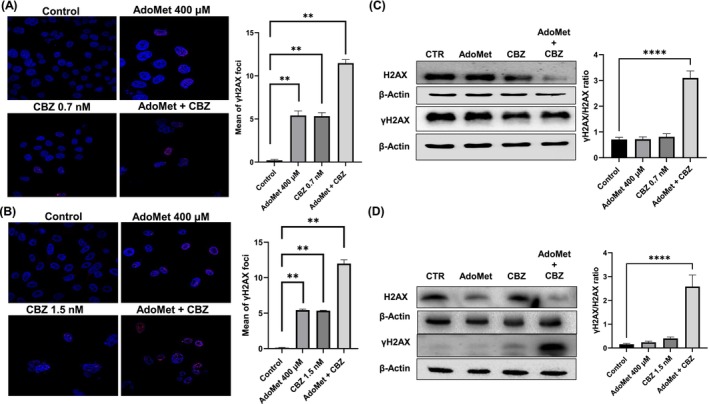
Effect of AdoMet, CBZ and their combination on DNA damage response in mCRPC cells. (A) DU 145 and (B) PC‐3 cells were cultured for 72 h in medium supplemented or not (control) with AdoMet 400 μM and CBZ 0.7 nM and 1.5 nM, respectively, alone or in combination. The images reported the representative immunofluorescence staining for phosphorylated γH2AX (red) in cells treated or not (control) with AdoMet and CBZ alone or in combination. Hoechst 33258 was used for nuclear staining (blue). Scale bar: 20 μM. About 100 nuclei for each group were scored in each experiment, and a threshold of five foci per cell was considered positive and reported in the histograms as a mean of γH2AX. Values represent the means of three experiments ± SD (***p < 0.001). The protein contents of γH2AX/H2AX in (C) DU 145 and (D) PC‐3 were detected by Western blot analysis using the total cell lysates. The house‐keeping protein β‐Actin was used as loading control. The graph shows the relative densitometric analysis, expressed as percentage of untreated control (100%). Error bar represents the standard deviation, ****p < 0.0001 versus untreated cells. The images are representative of three immunoblotting analyses that were obtained from three independent experiments.

Free radicals are highly reactive and can cause non‐specific injury to biological macromolecules, such as lipids, proteins, and nucleic acids [33]. Therefore, a DNA damage assay was performed to investigate the potential involvement of AdoMet‐ and CBZ‐induced ROS in causing damage to DNA, ultimately leading to mCRPC cancer cell death. Our study evaluated the expression of γH2AX, the main responsive marker for DNA damage, using both immunofluorescence and Western blot assays. As reported in Figure 4A,B, the immunofluorescence staining of mCRPC cells revealed an increase in the intensity of the γH2AX signal in the nuclei of AdoMet‐ and CBZ‐treated cells, indicating the development of DNA damage foci. The accumulation of DNA damage was particularly pronounced in cells treated with the drug combination, confirming the synergistic effect of the two drugs. These findings were further corroborated by performing Western blot analysis which exhibited an increased γH2AX/H2AX ratio, as suggested in Figure 4C,D.

Collectively, these findings suggest that the redox imbalance induced by the AdoMet and CBZ treatment can induce significant DNA damage in mCRPC.

### 3.6 AdoMet and CBZ Effect on Mitotic Spindle Assembly

**FIGURE 5:**
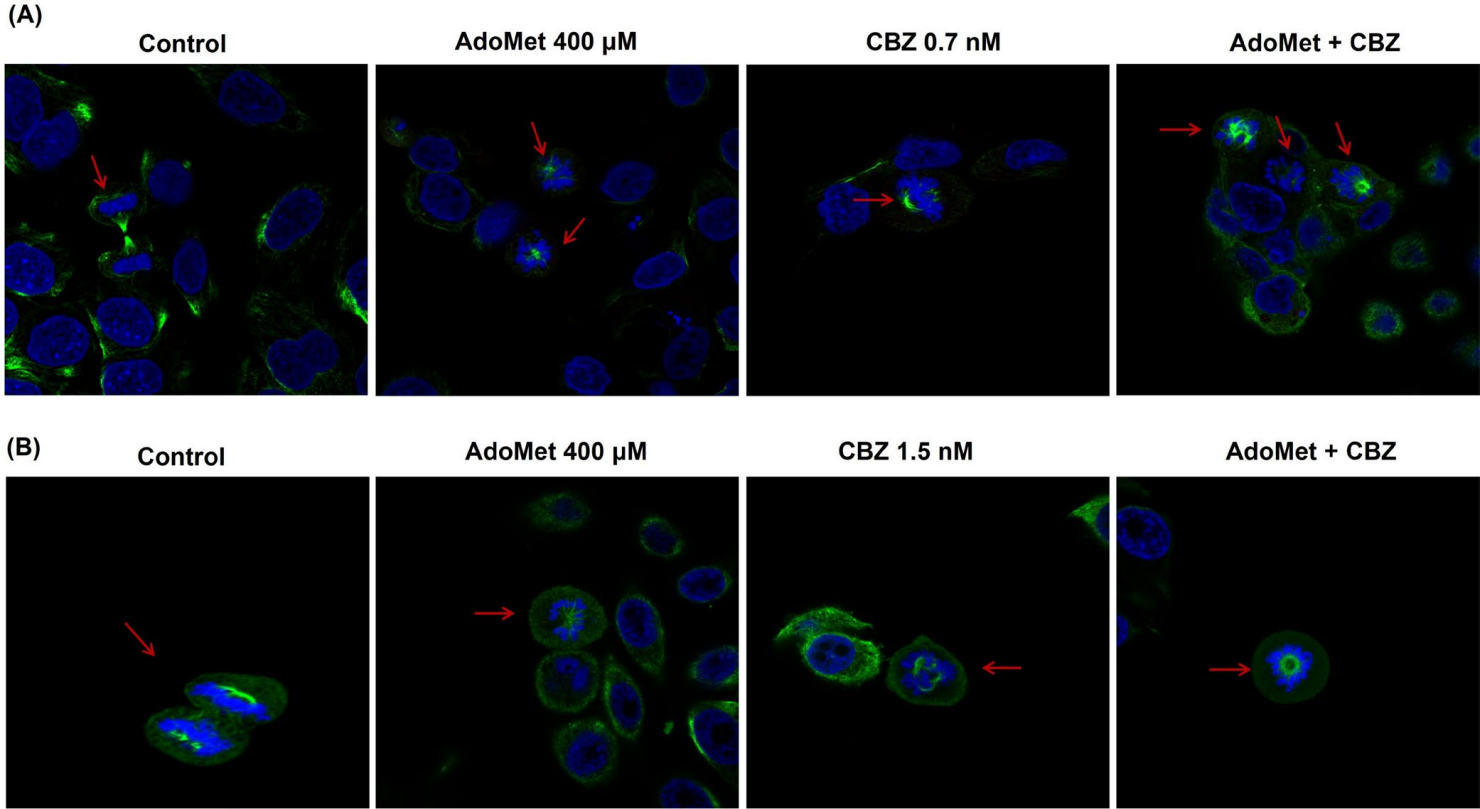
Effect of AdoMet, CBZ and their combination on mitotic microtubule and spindle organization in mCRPC cells. Representative immunofluorescence images (A) DU 145 and (B) PC‐3 treated or not (control) with 400 μM of AdoMet and 0.7 nM and 1.5 nM of CBZ, respectively, alone or in combination. Mitotic tubulin was immunostained with antibody anti *α*‐tubulin (green). Hoechst 33258 was used for nuclear staining (blue). Images are representative of three independent experiments. Scale bar 20 μm. As shown, the treated cells exhibited missegregated chromosomes, decondensed chromatin, and the absence of the spindle (*α*‐tubulin, green), above all in the combined treatment, indicative of mitotic catastrophe.

Mitotic catastrophe is defined as a dysregulated mitotic event, characterized by aberrant chromosome configurations and altered spatial rearrangements. It is considered an onco‐suppressive mechanism, induced by chemical stress, DNA damage, and chemotherapeutic agents, which makes cancer cells unable to complete mitosis and directs them to a permanent growth‐arrested state leading to cell death. Recently, we demonstrated that AdoMet can induce mitotic catastrophe in glioblastoma cells, leading to apoptosis [34]. Based on this, we analyzed the morphology of mCRPC cells following treatment with AdoMet and/or CBZ by immunofluorescence staining with *α*‐tubulin antibody to assess microtubules and the mitotic spindle, and with Hoechst 33,258 to stain the chromosome. As illustrated in the confocal pictures in Figure 5A,B, untreated cells in mitosis displayed correctly assembled bipolar spindle structure, with chromosomes (blue) exhibiting correct alignment at the metaphase plate. In contrast, cells treated with AdoMet and CBZ in mitosis exhibit disarranged chromosomes (blue) and a lack of microtubule mitotic machinery (green) (Figure 5A,B). Moreover, this disorganization of the mitotic spindle was more pronounced when AdoMet and CBZ were used in combination. These findings indicate that AdoMet induces alteration of the morphology in mCRPC cells, indicating the induction of mitotic catastrophe.

### 3.7 AdoMet and CBZ Synergistically Induce Apoptosis in mCRPC Cells

**FIGURE 6:**
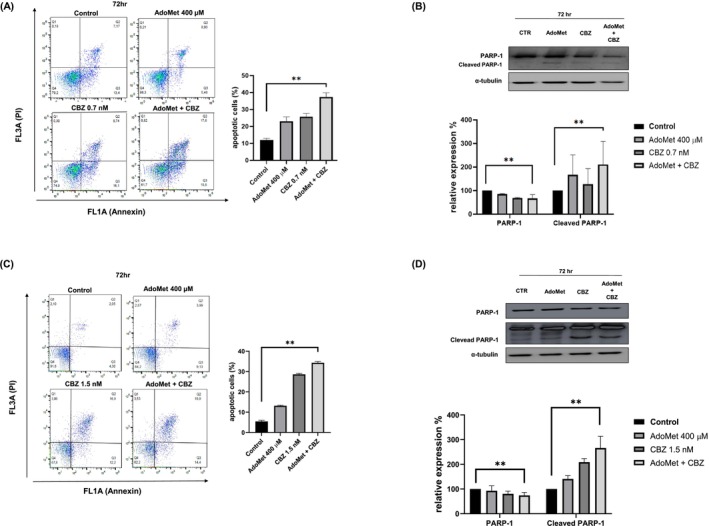
Effect of AdoMet, CBZ and their combination on apoptosis in mCRPC cells. (A) DU 145 was treated with 400 μM AdoMet and 0.7 nM CBZ and (C) PC‐3 was treated with 400 μM AdoMet and 1.5 nM CBZ, alone or in combination for 72 h. Apoptosis was evaluated by FACS analysis. Representative dot plots of both Annexin V‐FITC and PI‐stained cells. The different quadrants show the percentage of cells: Viable cells, lower left (Q4); early apoptotic cells, bottom right (Q3); late apoptotic cells, top right (Q2); non‐viable necrotic cells, upper left (Q1). For each sample, 2 × 10^4^ events were acquired. Analysis was carried out by triplicate determination of at least 3 separate experiments. Data represents the average of three independent experiments. The protein levels of PARP‐1 in (B) DU 145 and (D) PC‐3 were detected by Western blot analysis using total cell lysates. The house‐keeping protein *α*‐tubulin was used as loading control. All data represents the average of three independent experiments. The images are representative of three immunoblotting analyses obtained from three independent experiments ± SD (**p < 0.01).

The activation of apoptotic pathways in mCRPC cells in response to treatment with 400 μM AdoMet and 0.7 nM CBZ (Figure 6A) or 400 μM AdoMet and 1.5 nM CBZ (Figure 6C), alone or in combination, for 48 (see File S1) and 72 h in DU 145 and PC‐3 cells, respectively, was analyzed using flow cytometry. As illustrated in Figure 6A,C, AdoMet and CBZ triggered apoptosis in DU 145 and PC‐3 cells after 48 and 72 h of treatment, exhibiting a more pronounced apoptotic response observed at later time points. Notably, the combined treatment showed a synergistic effect in inducing cell death, with apoptosis percentages of approximately 25% and 15% at 48 h and 37% and 34% at 72 h, respectively, in DU 145 and PC‐3 cells, showing a significant increase compared to the control cells.

To verify these observations, PARP‐1 cleavage, a hallmark of apoptosis induced by AdoMet and/or CBZ, was assessed by Western blot. As illustrated in Figure 6B,D, exposure to each drug alone caused cleavage of the PARP‐1 protein, which was significantly increased in the combined treatment, confirming the synergistic effect of AdoMet and CBZ to activate the apoptotic process.

## 4 Discussion

In our study, we demonstrate that the combination of CBZ and AdoMet is more effective than either agent alone in enhancing the antitumor effects in mCRPC cells, focusing on DU 145 and PC‐3. A key advantage of natural compounds, such as AdoMet, lies in their ability to selectively induce tumor cell death while sparing normal cells from significant cytotoxic effects under comparable experimental conditions [35]. Although AdoMet is known to influence both normal and cancer cells, the differential transcriptional landscapes in these cell types lead to distinct phenotypic outcomes. Indeed, in cancer cells, AdoMet often triggers anti‐proliferative and pro‐apoptotic responses, while normal cells remain largely unaffected.

Given its established clinical use for conditions such as mood disorders, fibromyalgia, and joint pain, AdoMet represents an attractive candidate for drug repurposing in oncology. The FDA has authorized its use as a dietary supplement and for the treatment of these conditions [28, 36, 37], and at pharmacological doses it demonstrates a safety profile characterized by a low incidence of adverse outcomes. With its well‐characterized pharmacokinetics and tolerability, AdoMet can potentially be advanced as a chemotherapy adjuvant more efficiently and cost‐effectively than a novel compound [19, 38, 39]. This approach not only reduces the time and expense of preclinical and early clinical development but also facilitates the rapid translation of preclinical findings into patient‐relevant applications, highlighting its potential as a readily implementable adjunct to existing cancer therapies. Our data demonstrate that AdoMet and CBZ treatment, alone or in combination, in mCRPC cells leads to a marked increase of ROS generation and accumulation, ultimately disrupting redox homeostasis and subsequent cancer cell death. Elevated levels of ROS have been detected in almost all types of cancer, where they promote several aspects of tumor initiation, progression, and metastasis. Cancer cells typically preserve ROS accumulation at moderately to highly elevated levels, over cytostatic yet under cytotoxic thresholds, through upregulation of their intrinsic antioxidant defense mechanisms. This subtoxic ROS environment not only supports tumor cell survival but also promotes proliferation, as ROS function as key signaling molecules in oncogenic pathways [40]. However, when exposed to additional ROS‐induced cellular stress, tumor cells become increasingly susceptible to damage caused by ROS. Increase ROS production or accumulation disrupt the redox balance triggering cellular cancer death.

Compound obtained from natural sources, such as polyphenols, can exhibit antioxidant activity that protects normal cells from oxidative damage, while they may function as oxidative agents, increasing intracellular oxidative stress and causing cancer cell [41]. In this study, we demonstrated that the ROS production and accumulation following AdoMet and CBZ treatment, alone or in combination, in mCRPC cells was induced by the strong reduction in the antioxidant intracellular defense. In cancer cells, the high ROS accumulation is often associated with a significant reduction in intracellular GSH levels. It has demonstrated a deficiency in GSH, disrupted GSH/GSSG ratio, and improved cellular susceptibility to oxidative stress. GSH is a key contributor to resistance to chemotherapy, and its repression employed in combined treatments has been demonstrated to significantly enhance chemotherapy effectiveness [30]. Notably, the reliance of cancer cells on robust antioxidant systems to manage elevated ROS levels makes them particularly vulnerable to strategies that disrupt redox homeostasis [42]. Consequently, targeting GSH synthesis or availability emerges as a promising therapeutic strategy to sensitize tumor cells and enhance the efficacy of chemotherapeutic treatments. In the work of Ye Hu et al., the quercetin and oxaliplatin combined treatment synergistically decreased intracellular GSH levels, increased ROS production, and reduced cell viability compared to oxaliplatin treatment alone in human colorectal HCT116 cancer cells [43]. GSH also plays a critical role in maintaining GPX4 activity, highlighted in our experimental model, in which the application of AdoMet and CBZ resulted in a marked reduction of GPX4 and catalase levels, two key antioxidant enzymes involved in counteracting oxidative stress, preserving redox homeostasis, and protecting cells from ROS‐mediated damage [44]. GPX4 oxidizes GSH to reduce hydrogen peroxide, organic hydroperoxides, and lipid hydroperoxides, exerting a protective antioxidant effect. A decrease in GSH levels, as well as reduced GPX4 expression, causes a decrease in intracellular antioxidant defense, resulting in an increase of lipid‐derived ROS and damage caused by oxidative stress. GPX4 overexpression represents a crucial component of the cellular antioxidant system, and the use of direct inhibitors of GPX4 expression or activity has been proposed as a promising strategy to suppress tumor progression both in cell culture and animal models [45].

Furthermore, reduced catalase expression in mCRPC cells led to increased sensitivity to hydrogen peroxide, as well as decreased proliferative and migratory abilities. Tumors with catalase deficiency were considerably relative to wild‐type tumors, while restoration of catalase function rescued the tumor progression rate [46]. The indicated findings suggest that inhibiting catalase could be a favorable therapeutic approach for selectively eliminating tumor cells. Due to elevated ROS levels in tumor cells, extensive damage to cellular macromolecules occurs, most notably to DNA.

ROS‐induced DSBs represent the most damaging lesions that trigger mutations inducing genomic instability and increasing sensitivity to cancer [31]. It has been reported that isoalantolactone, an isomeric sesquiterpene lactone extracted from *Inula helenium* , markedly enhanced doxorubicin‐induced cell cytotoxicity in colorectal carcinoma cells by ROS‐induced damage to DNA activating the JNK signaling pathway [47]. Our findings demonstrated that in mCRPC cells, AdoMet and CBZ treatment, alone or in combination, lead to antioxidant intracellular defense depletion and a marked increase of intracellular ROS, resulting in significant damage to DNA. This is evidenced by the formation of foci at DSB sites, shown by increased levels of the H2AX phosphorylated form, a well‐established marker of DNA DSBs.

Irreversible DNA damage is a critical driver of tumor cell apoptosis and plays a central role in the mechanism supporting cancer cell death. Moreover, based on our last study [34] that demonstrated the AdoMet capacity to influence the cell division by affecting the microtubule formation and inducing DNA damage, we have also evaluated the spindle formation in DU 145 and PC‐3 cells. Microtubules are cytoskeletal filaments constituted by *α*‐ and β‐tubulin heterodimers that play a main function in maintaining cell morphology, cell division, cell signaling, and vesicle trafficking. CBZ interacts with the N‐terminal region of the β‐tubulin subunit, enhancing microtubules polymerization [5]. This binding impairs the assembly of mitotic spindles, resulting in cell cycle arrest with subsequent inhibition of cell division. Moreover, several genotoxic chemotherapeutic agents and radiotherapy directly target cellular DNA, inducing primary damage that contributes to the onset of mitotic catastrophe. Our results confirm the ability of AdoMet to interfere with mitotic spindle assembly, thereby enhancing the antimitotic action of CBZ.

AdoMet and CBZ have shown the ability to promote apoptosis in mCRPC cells, particularly in the combination treatment. The AdoMet ability to enhance the pro‐apoptotic effects of various anticancer agents has been widely demonstrated in the literature across multiple types of human tumors. In breast cancer cells, such as MDA‐MB‐231, AdoMet potentiates 5‐azacytidine‐induced inhibition of uPA gene, a key promoter of tumor metastasis, reducing invasiveness [27]. In cervical cancer, AdoMet, in combination with selenium compounds, reduces migration, adhesion, and proliferation by modulating AKT and ERK pathways [48]. Furthermore, AdoMet synergizes with doxorubicin in CG5 breast cancer cells through Fas/FasL activation [49], while in MCF‐7 cells, co‐treatment with chloroquine promotes apoptotic cell death [50]. In pancreatic cancer, AdoMet enhances gemcitabine‐mediated apoptosis and suppresses invasion via inhibition of the JAK2/STAT3 pathway. Similarly, AdoMet augments cisplatin‐induced apoptosis and inhibits migration in head and neck squamous carcinoma cells. In colorectal cancer models, AdoMet boosts 5‐fluorouracil efficacy by suppressing autophagy, a protective response to chemotherapy [51]. Finally, in breast cancer xenografts, AdoMet co‐administered with decitabine significantly reduces tumor burden and metastasis [52].

Our findings confirm that AdoMet enhances tumor cell sensitivity to chemotherapeutic agents by attenuating resistance mechanisms typically activated in response to standard treatments. This chemosensitizing effect translates into improved therapeutic efficacy, as evidenced by reduced tumor growth and enhanced apoptotic response under combinatorial regimens. In conclusion, we confirm in this study the pleiotropic effect of AdoMet in promoting tumor cell death by modulating both ROS production and accumulation, as well as interfering with mitotic spindle assembly, thereby enhancing the antitumoral action of CBZ and demonstrating that the combined treatment of AdoMet with CBZ significantly promotes antitumoral activity in mCRPC cells compared to CBZ alone. The current findings support the potential of AdoMet as a chemosensitizer, improving the positive efficacy of CBZ in PCa. Given AdoMet's favorable safety profile, this combination therapy offers a potential approach to overcoming drug resistance in advanced PCa, warranting further investigation in clinical settings. The findings of this work are based on in vitro experiments. Further in vivo studies will be necessary to assess the therapeutic efficacy of the AdoMet and CBZ combination, potentially supported by advanced drug delivery systems. Advanced drug delivery systems using nanotechnology are increasingly being explored to improve the effectiveness of cancer treatments by enhancing drug solubility, stability, and bioavailability, while reducing systemic exposure and toxicity. Nanoparticles are especially useful because they can protect sensitive drugs, control their release, and promote passive or active tumor targeting, ensuring that higher amounts of the therapeutic agent reach cancer tissue. Nanoparticle formulations can also modulate drug biodistribution and tumor‐site accumulation, helping to achieve a better balance between therapeutic efficacy and toxicity. Although nanoformulations are generally more expensive than the use of free natural products, these costs are balanced by important advantages, including improved target specificity, enhanced biological activity with fewer side effects, prolonged half‐life, and sustained drug release. Evidence from PCa research clearly supports this approach: nanoparticle formulations of curcumin significantly improve its poor solubility, instability, and low pharmacokinetic profile, while quercetin nanomicelles show increased stability, drug loading capacity, and intracellular distribution in prostate cancer models. Similar nanotechnological strategies are currently being investigated for other natural compounds relevant to PCa, such as betulinic acid, capsaicin, sintokamide A, niphatenones A and B, and atraric acid, although further studies are needed to confirm their clinical value. In addition, established phytocompound‐derived anticancer agents, such as CBZ, may also benefit from advanced nanoparticle‐based formulations, potentially improving drug delivery and reducing systemic toxicity in future prostate cancer therapies [53]. Although only a limited number of studies have focused on the formulation of AdoMet‐loaded nanoparticles, available evidence indicates that nanocarriers can act as environmentally responsive delivery systems capable of stabilizing AdoMet and controlling its release profile [54, 55]. Building on these preliminary findings, the development of innovative nanovector‐based technologies incorporating AdoMet, either alone or in combination with chemotherapeutic agents, may represent a promising strategy to improve its bioavailability, reduce off‐target effects, and enable selective delivery to PCa cells. Such approaches deserve further investigation and may provide a solid foundation for future translational and clinical applications in the management of PCa.

## Author Contributions

**Francesca Cadoni:** performed the experiments, prepared the figures; **Roberta Arpino:** performed the experiments and prepared the figures; **Vitale Del Vecchio:** software and FACS analysis. **Laura Coppola:** performed the immunofluorescence experiments; **Cristina Pagano:** performed the immunofluorescence experiments data curation. **Aditya Nigam:** software and FACS analysis; **Laura Mosca:** conceived the study, designed the experiments; and **Marina Porcelli:** contributed to the writing and critical review of the manuscript.

## Funding

The authors have nothing to report.

## Conflicts of Interest

The authors declare no conflicts of interest.

## Supporting information


**Data S1:** Supporting Information.

## Data Availability

The data that support the findings of this study are available from the corresponding author upon reasonable request.
