# Supplementary material for: Improved Chemosensitivity in Metastatic Castration‐Resistant Prostate Cancer: The Synergistic Effects of S‐Adenosylmethionine and Cabazitaxel
Source: Cancer Med. 2026 Apr 13;15(4):e71784. doi: 10.1002/cam4.71784 (PMC13072059; doi:10.1002/cam4.71784)
Supplement: Supplementary file 1 — Data S1: Supporting Information. [file CAM4-15-e71784-s001.pdf]

DU 145

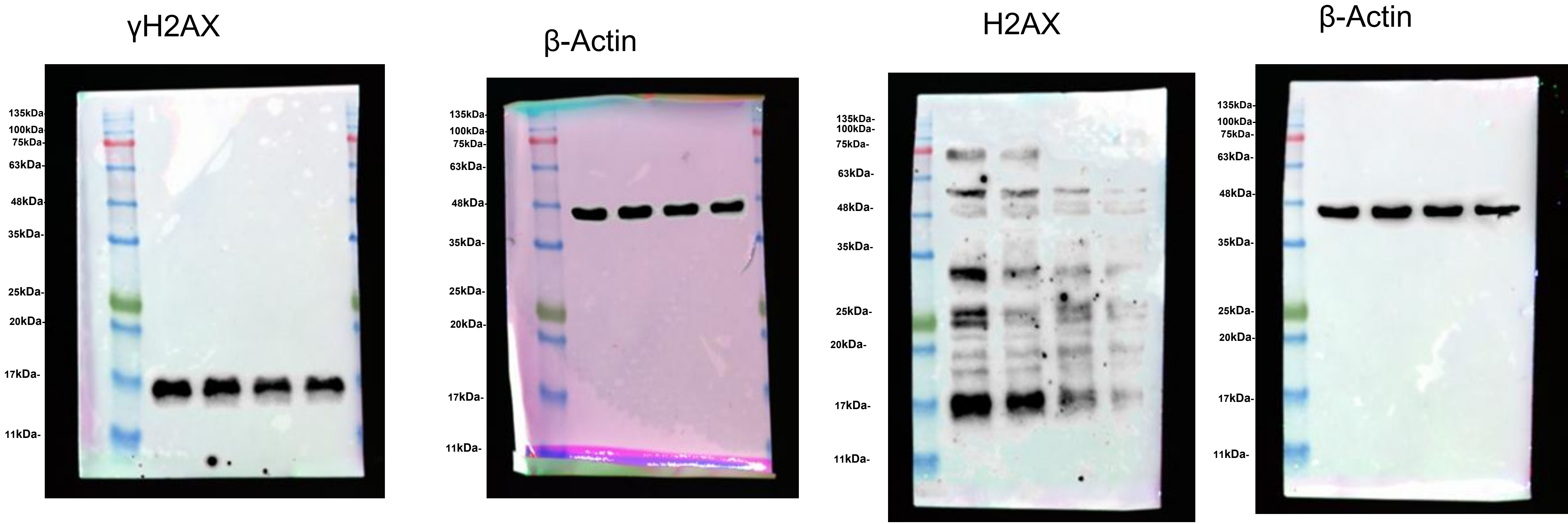
γH2AX
β-Actin
H2AX
β-Actin
135kDa-
100kDa-
75kDa-
63kDa-
48kDa-
35kDa-
25kDa-
20kDa-
17kDa-
11kDa-

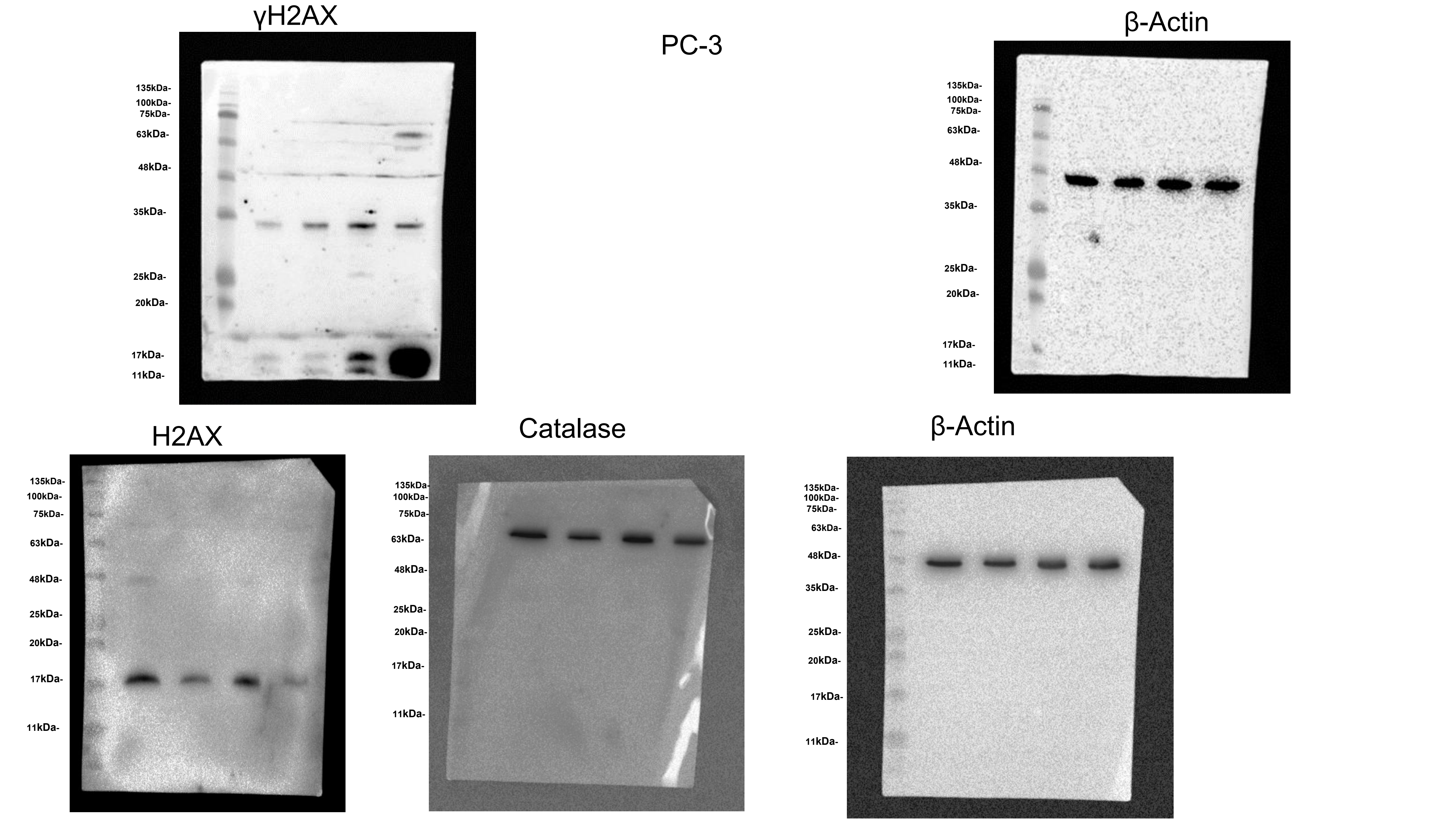
γH2AX
PC-3
β-Actin
135kDa-
100kDa-
75kDa-
63kDa-
48kDa-
35kDa-
25kDa-
20kDa-
17kDa-
11kDa-
H2AX
Catalase
β-Actin

DU 145

PARP-1

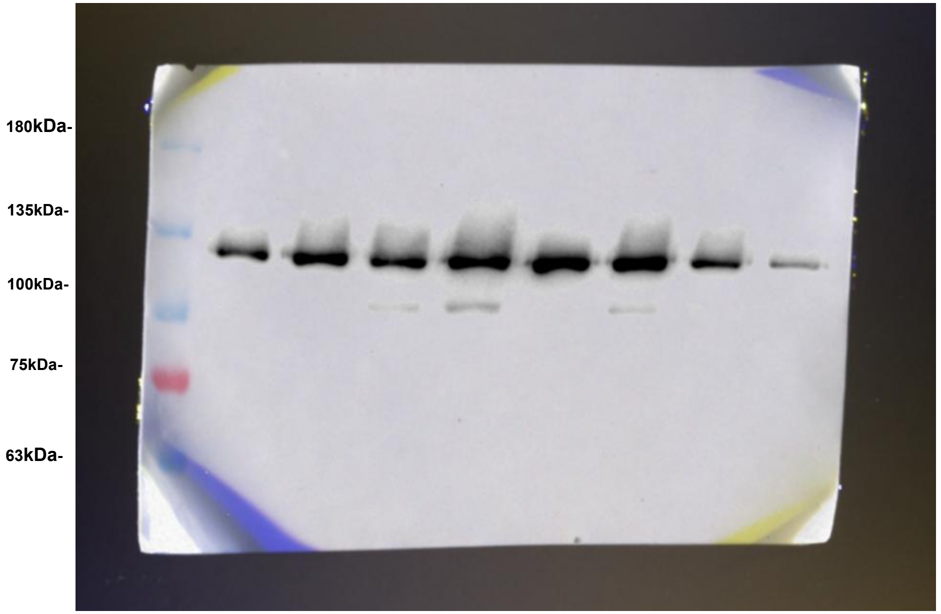

α-tubulin

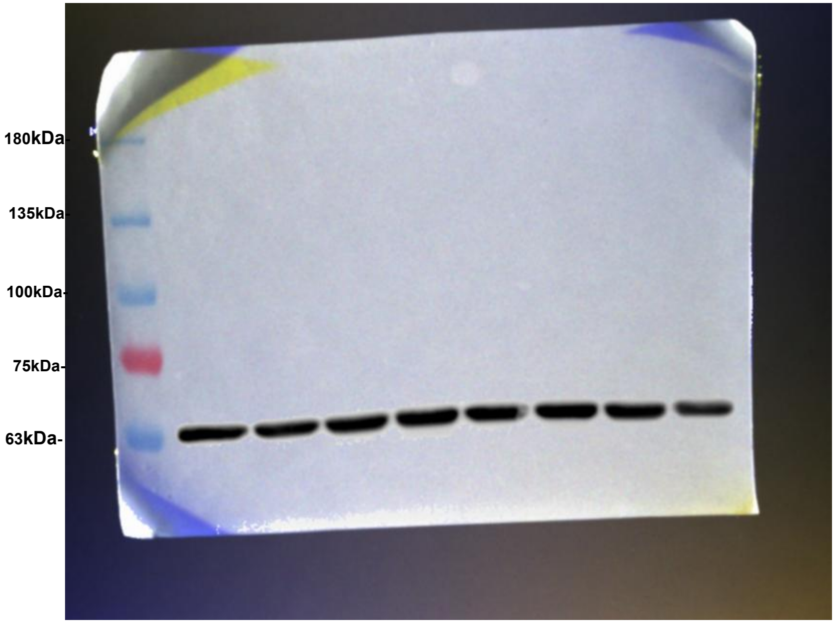

PC-3

PARP-1

α-tubulin

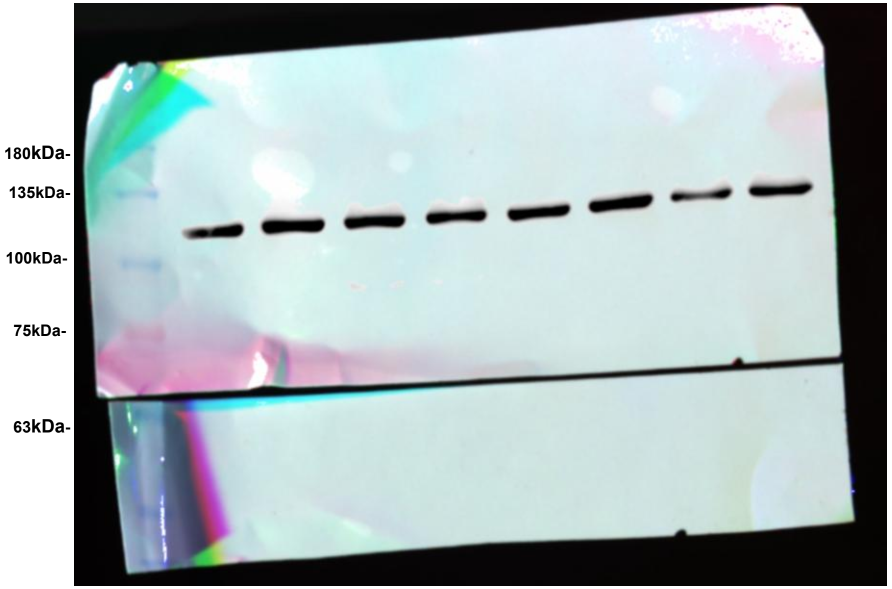

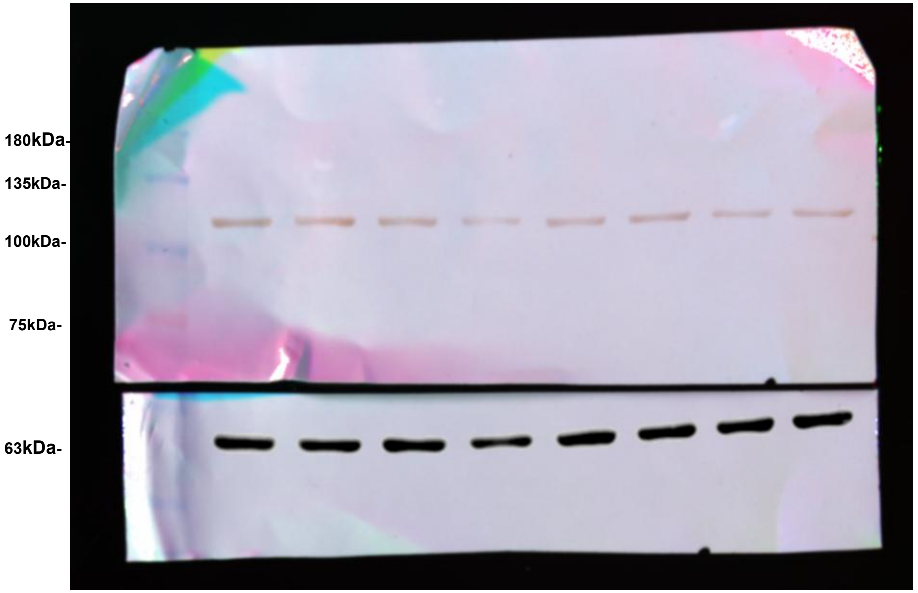

DU 145

GPX4

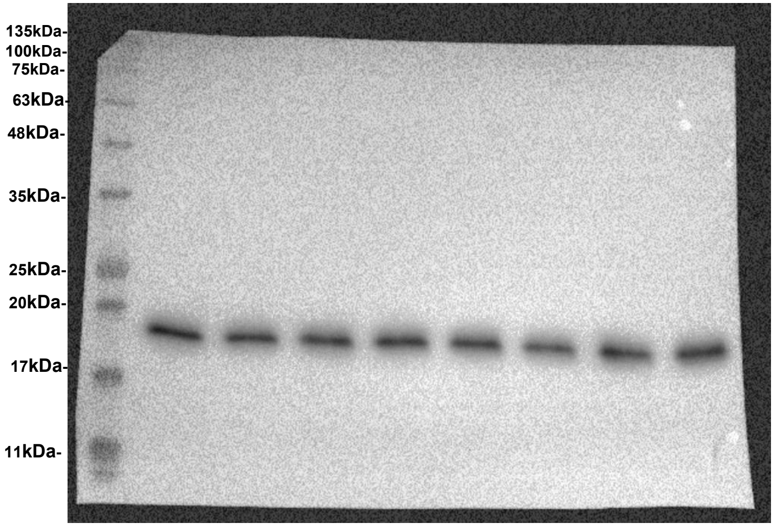

Catalase

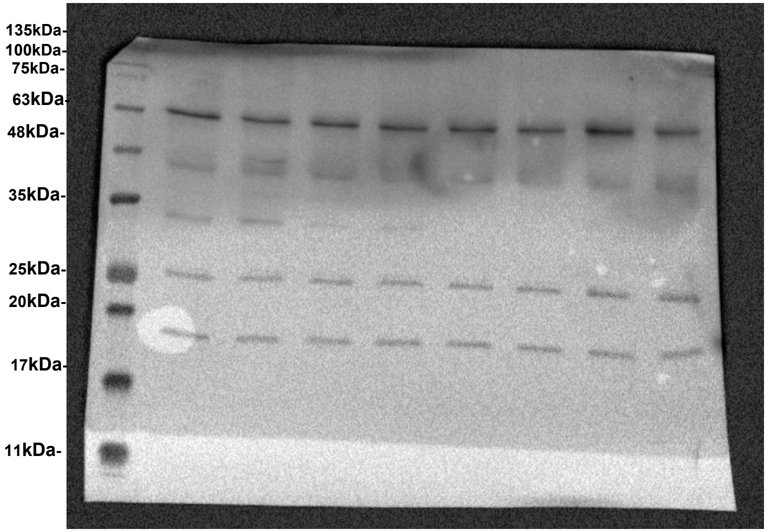

β-actin

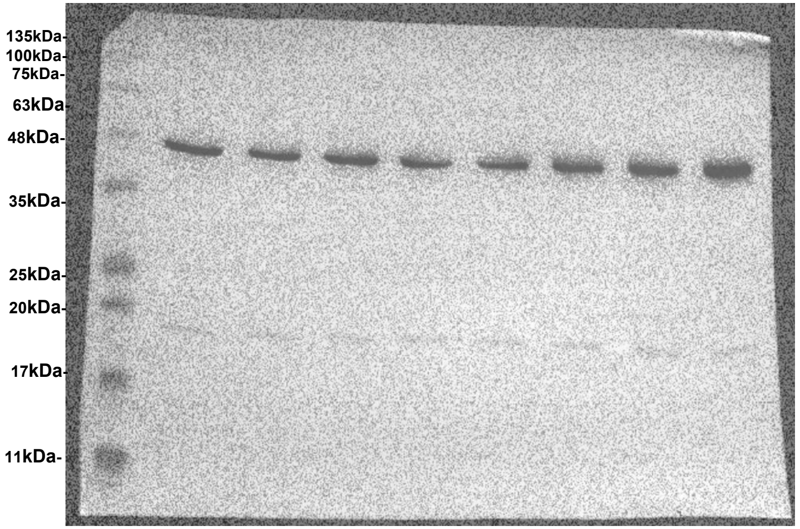

PC-3

GPX4

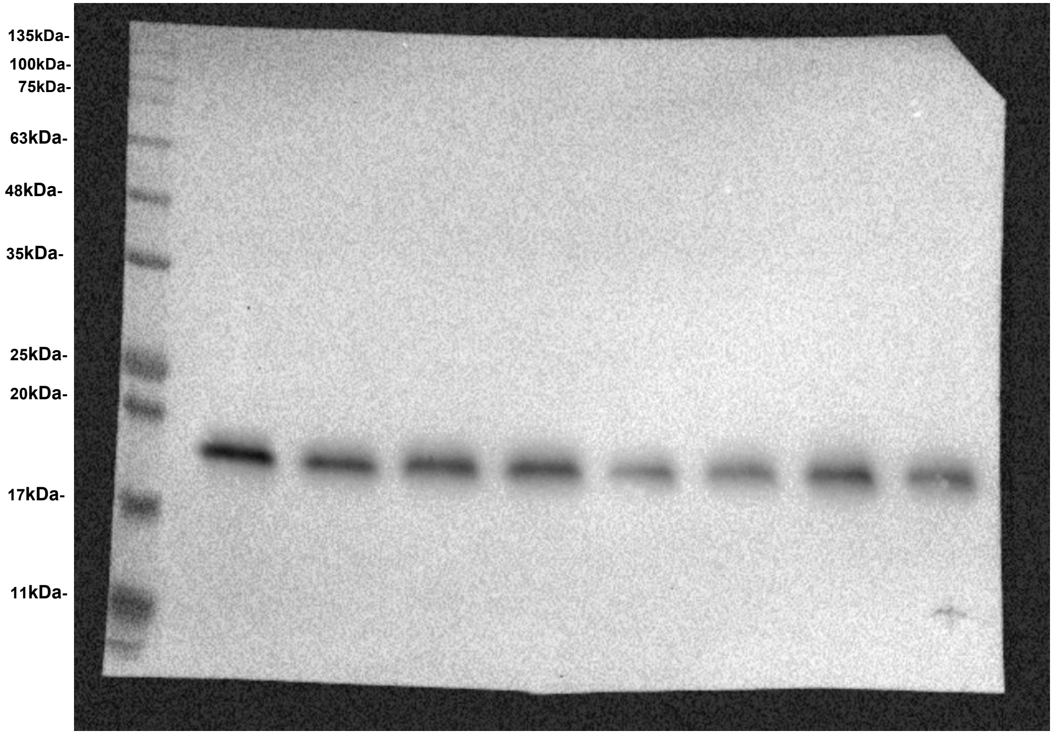

β-actin

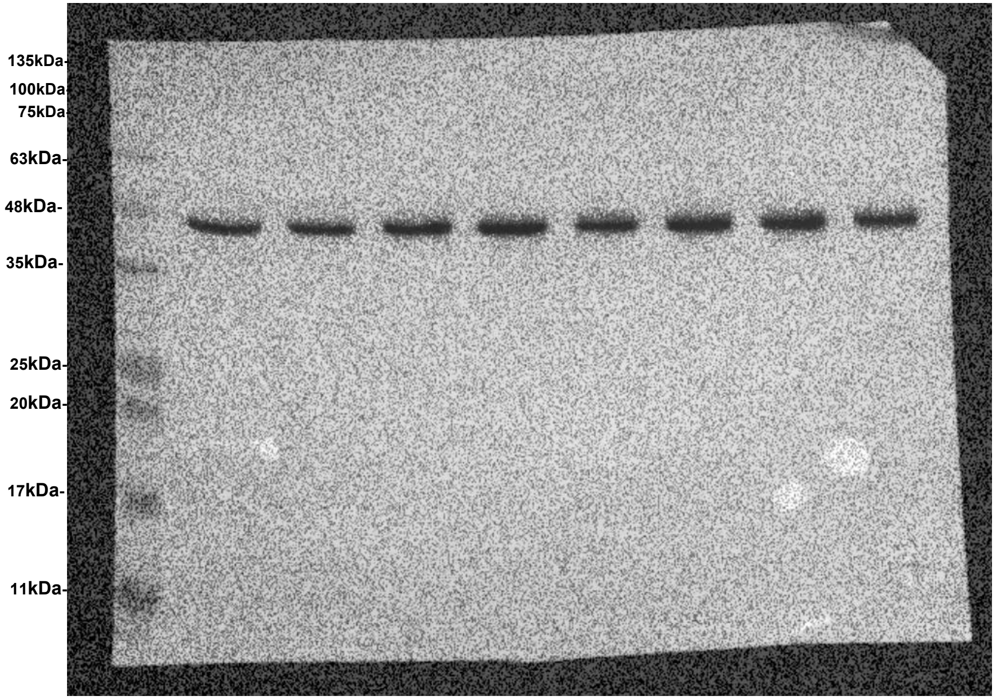

DU 145

(A)

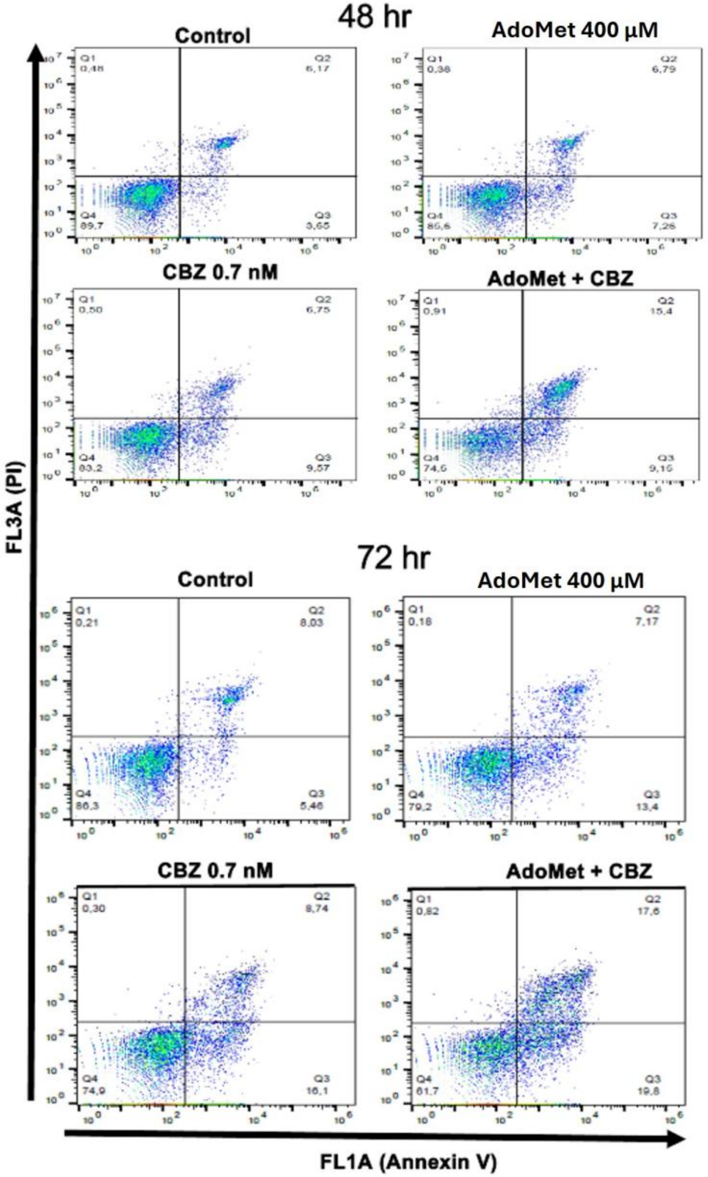
48 hr
Control
AdoMet 400 µM
CBZ 0.7 nM
AdoMet + CBZ
72 hr
Control
AdoMet 400 µM
CBZ 0.7 nM
AdoMet + CBZ
FL3A (PI)
FL1A (Annexin V)

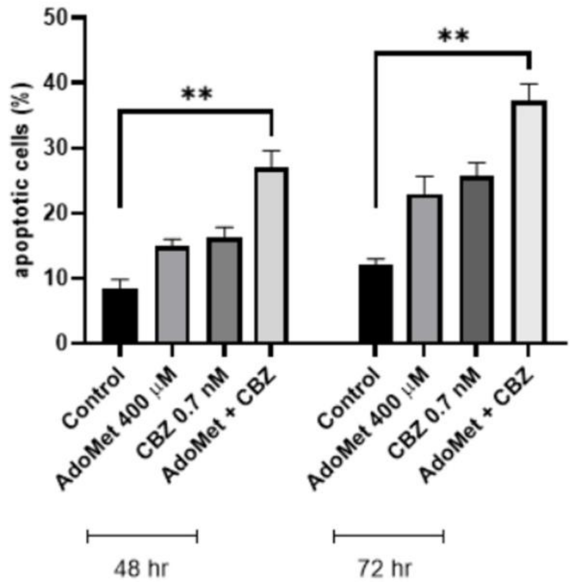
apoptotic cells (%)
**
**
Control
AdoMet 400 µM
CBZ 0.7 nM
AdoMet + CBZ
Control
AdoMet 400 µM
CBZ 0.7 nM
AdoMet + CBZ
48 hr
72 hr

(C)

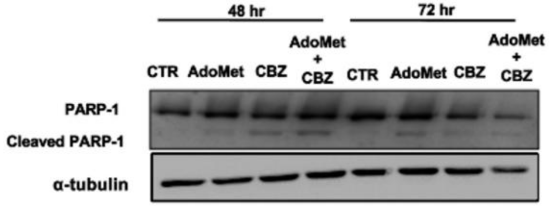
48 hr
72 hr
CTR
AdoMet
CBZ
AdoMet + CBZ
CTR
AdoMet
CBZ
AdoMet + CBZ
PARP-1
Cleaved PARP-1
α-tubulin

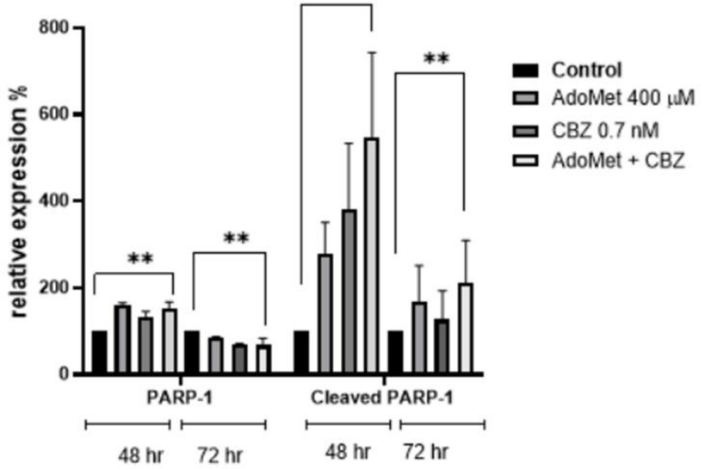
relative expression %
**
**
**
**
Control
AdoMet 400 µM
CBZ 0.7 nM
AdoMet + CBZ
PARP-1
Cleaved PARP-1
48 hr
72 hr
48 hr
72 hr

PC-3

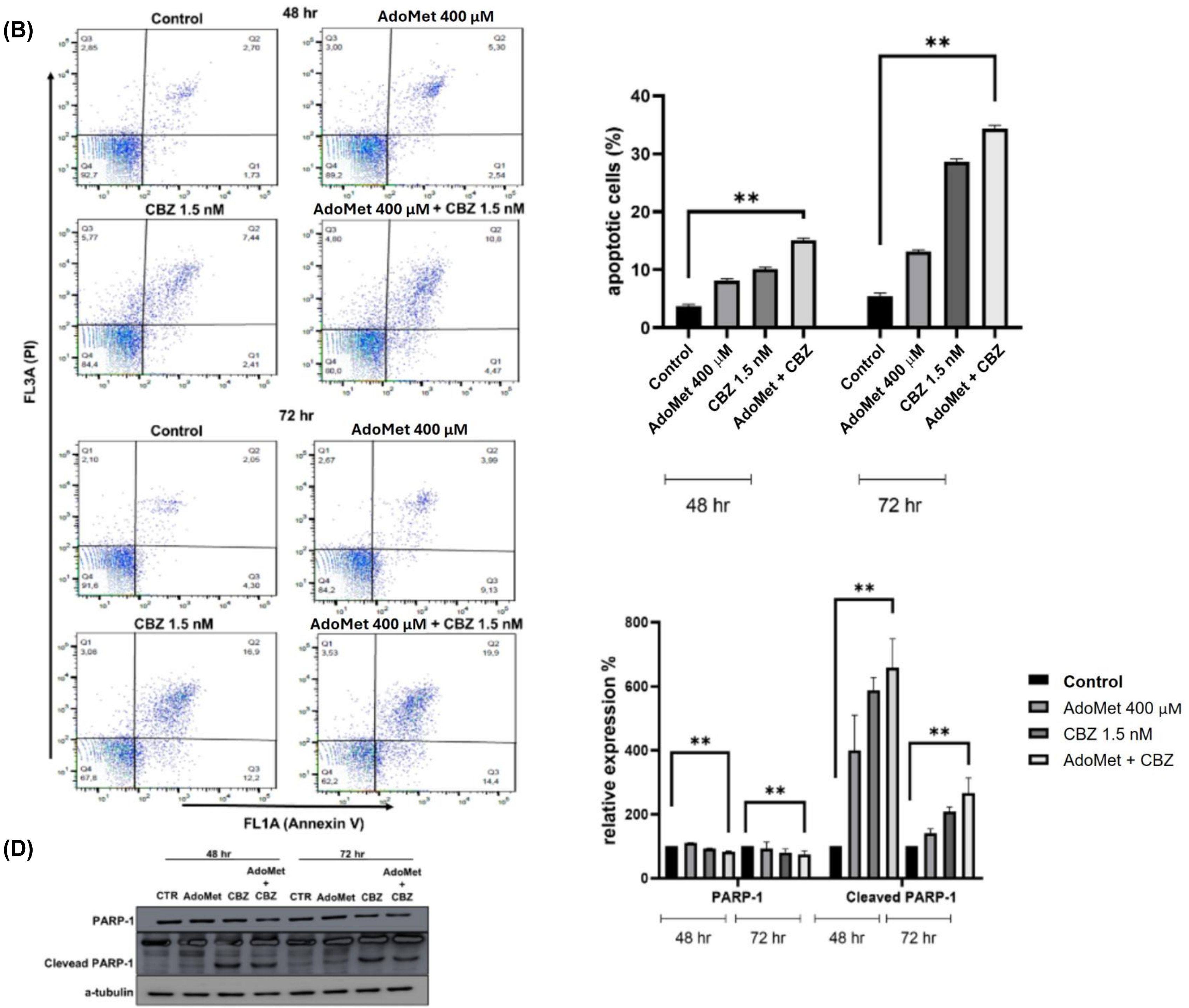

(B)
48 hr
Control
AdoMet 400 μM
CBZ 1.5 nM
AdoMet 400 μM + CBZ 1.5 nM
72 hr
FL3A (PI)
FL1A (Annexin V)
apoptotic cells (%)
Control
AdoMet 400 μM
CBZ 1.5 nM
AdoMet + CBZ
48 hr
72 hr
**
(D)
CTR
AdoMet
CBZ
AdoMet + CBZ
PARP-1
Clevead PARP-1
a-tubulin
relative expression %
PARP-1
Cleaved PARP-1
Control
AdoMet 400 μM
CBZ 1.5 nM
AdoMet + CBZ
